# Analysis of ECG-based arrhythmia detection system using machine learning

**DOI:** 10.1016/j.mex.2023.102195

**Published:** 2023-04-20

**Authors:** Shikha Dhyani, Adesh Kumar, Sushabhan Choudhury

**Affiliations:** Department of Electrical and Electronics Engineering, School of Engineering, University of Petroleum & Energy Studies, Dehradun 248007, India

**Keywords:** Arrhythmia detection, Heart rate, RR interval, Deep learning, Residual neural network, ECG signals, SVM, 3-D wavelet transform

## Abstract

The 3D Discrete Wavelet Transform (DWT) and Support Vector Machine (SVM) are used in this study to analyze and characterize Electrocardiogram (ECG) signals. This technique consists of three stages: ECG signal preprocessing, feature extraction, and ECG signal order. The 3D wavelet transform is a signal preprocessing technique, de-noising, along with wavelet coefficient extraction.

• SVM is used to categorize the ECG through each of the nine heartbeat types recognized by the various classifiers. For this work, around 6400 ECG beats were looked at over the China Physiological Signal Challenge (CPSC) 2018 arrhythmia dataset.
• The best degree of exactness was acquired when level 4 rough constants with Symlet-8 (Sym8) channel were utilized for arrangement. Utilizing the ECG signals from CPSC 2018 data set, the SVM classifier has a normal precision of 99.02%, which is much better than complex support vector machine (CSVM) 98.5%, and weighted support vector machine (WSVM) 99%.
• The suggested approach is far superior to others in terms of accuracy, and classification of several diseases of arrhythmia.

## Graphical abstract

**Figure:**
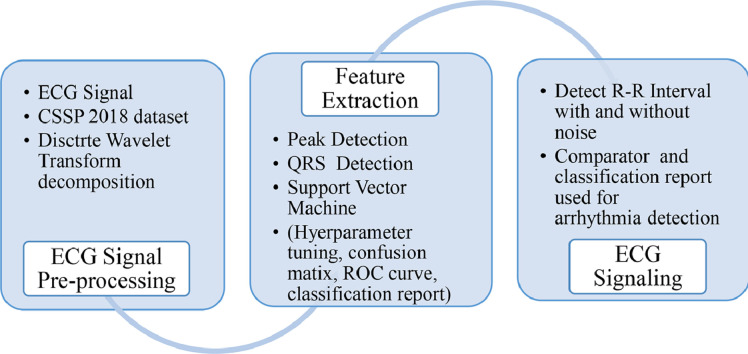

Specifications table

**Table:** 

Subject area:	Computer Science and Electronics
More specific subject area:	Machine Learning
Name of your method:	3-D wavelet transform
Name and reference of original method:	Automatic ECG arrhythmia classification using dual tree complex wavelet based features, *AEU-International Journal of Electronics and Communications*, *69*(4), 2015, 715–721.
Resource availability:	Not Applicable

**Method Details**

## Background

**Fig. 1:**
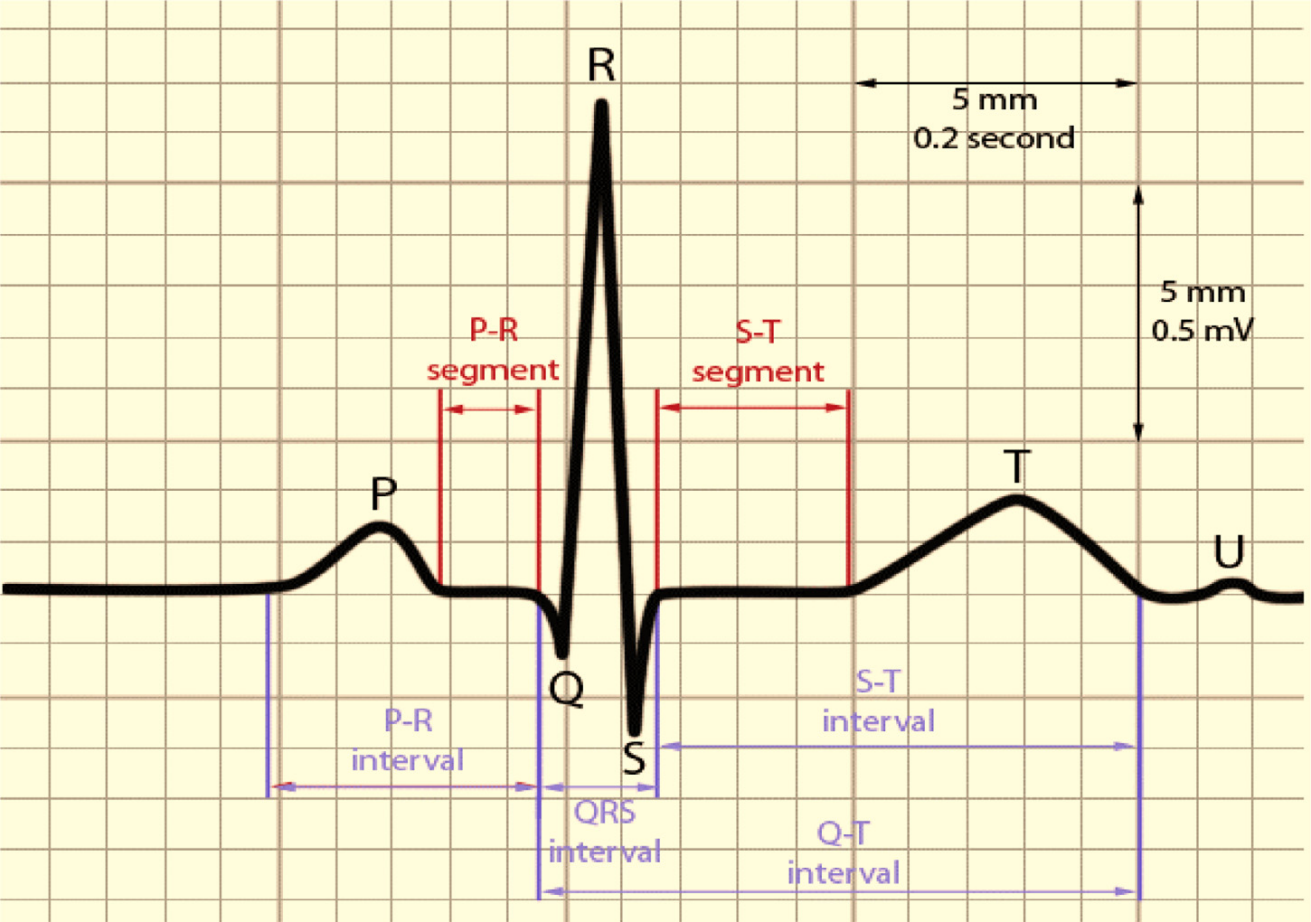
An ECG waveform.

On an overall worldwide basis, cardiovascular diseases are the essential driver of mortality incapacity. It is a primary source of mortality incapacity, with significant ramifications for individuals well-being. The World Health Organization (WHO) just opened up the best ten worldwide well-being concerns for 2019. Coronary illness is a commonplace non-irresistible infection on the rundown. Because of the trouble in restoring, early screening treatments are fundamental. An ECG is an essential instrument that can consistently record the heart's electrical movement throughout some time. In clinics around the world, there are more than 300 million clinical ECG records. ECG is the most essential, helpful, prudent routine assessment approach. It is ordinarily done for clinical screening of numerous cardiovascular infections, like passing judgment on arrhythmia, diagnosing myocardial schema, mirroring the heart's construction, and giving essential reference data to clinicians. With the rise of healthcare 4.0 Artificial Intelligence (AI) improvement, the significance of programmed findings has become progressively unmistakable. Mechanized ECG examination gives extra demonstrative data. It can screen heart circumstances for 24 h, which is also helpful for portable clinical distant analysis. The ECG is a heart's myocardium electrical movement recorded through one flow cycle. It is depicted by a repetitive grouping of P wave, QRS wave, and T waves, addressing the cadenced depolarization of the myocardium's repolarization identified with the atria ventricles inconsistencies. Over every heart cycle, Anodes are positioned on the body exterior to record the ECG. To get a broad picture of the heart's activity, a typical 12-lead system is used. ECG is quite possibly the most proficient analytic system for various coronary illnesses. The different components of the ECG signal like the PR span, QRS stretch, QT stretch, ST span, PR fragment, and ST-section are utilized to construe the cardiovascular state as depicted in Fig. 1. The great PC helped assessment techniques target isolating these time plane parts from digitized ECG information. Moreover, identifying the QRS edifice and the R-peak gives automated ECG [1] examination calculations basics.

The cardiovascular framework becomes more vulnerable to illness as individuals age [2]. Besides, the supply routes strengthen, and the substantial mass of the left ventricle thickens, diminishing muscle consistency and adversely debilitating capacity. Moreover, arrhythmias are bound to create because of the underlying electrical changes in the heart [3]. As an outcome, arrhythmias are unpredictable heartbeat rhythms. A part of these unusual rhythms is protected, yet others are hazardous. AFIB is the most well-known sort of arrhythmia, portrayed by the clumsy atrial movement brought about by developing a primary number of unusual foci that start electrical information sources autonomous of the SAN [4]. The AVN gets electrical contributions at unpredictable spans from the atria, and behaves them to the ventricles, bringing about a sporadic QRS complex heartbeat. When a drive does not cease to exist, later normal heart initiation proceeds to re-energize the heart known as reemergence. The more ectopic foci there are, the more impressive the shot at reemergence, which is the thing that drives the progress from AFIB eruptions to persistent AFIB [5,6]. At 150–220 beats each moment, the ECG mood of AFIB is turbulent fast. AFIB is characterized by a strange RR span, sporadic quick ventricular constriction, and the P wave's shortfall on the electrocardiogram. AFL befalls in a full-scale re-participant circuit have a distinctive electrophysiological system [7]. In the chamber the electrical circuit is round and quickly directing, bringing about an atrial fibrillation pace of 240 to 360 beats each moment. This review presents a specialized strategy for mechanizing multiclass arrhythmia distinguishing proof involving RR spans. This innovation arrangement takes the state of a sign healing framework that uses AI [8] to help clinical direction. Benchmark information was utilized to test the framework. During pre-healing, we certify that the preparation testing information came from the patient [9] gatherings that were fundamentally unrelated. This guarantees that test sets are autonomous and not seen during the learning system, contrasting our discoveries with the setup information.

## Method description

The techniques used to help our declaration that mechanized distinguishing proof of arrhythmias in RR stretch signs is attainable are portrayed in this part. In addition, the strategies were utilized to construct a sign-healing framework that utilizes benchmark information to test the Support Vector Machine (SVM) calculation. The information-healing framework is portrayed in Fig. 1 as a square graph. The planning of the accessible ECG [10,11] datasets is the initial phase in the healing. The RR stretch sign was then made by separating the beat-to-beat span. Next, these beat-to-beat span signals were examined to permit the SVM model to be prepared and tried. Finally, execution estimations created from a disarray framework ROC were utilized to evaluate the model. The following parts go over both the information and the healing techniques in more prominent profundity. With the assistance of SVM, we anticipated the class of the information into two sorts: normal and abnormal.

**Fig. 2:**
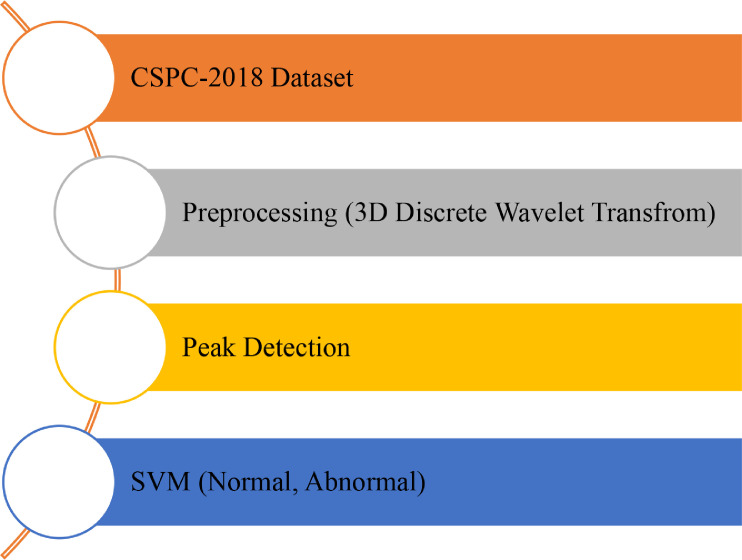
System model.

Fig. 2 shows one information base that sources the CPSC 2018 information to prepare and test the SVM model. The ECG information was denoised with the 3D wavelet change's assistance. The ECG accounts were gathered from 11 medical clinics as recorded in Table 1. The preparation set covers 6877 accounts (out of which male: 3699, female: 3178) using 12 leads ECG enduring from 6 s to only 60 s, and the test set covers 2954 ECG accounts with comparative lengths. Unfortunately, the test set is inaccessible to people who will stay private to score. ECG reports were assessed as 500 Hz. All information is given in MATLAB structure (individual recording is a. mat document containing the ECG information, just as the patient sex age data). There is a REFERENCE.csv document to prepare information to give the names of each recording. Most of the records only have one label (as indicated as the first label). The CPSC-2018 intends to energize the improvement of calculations to distinguish the beat/morphology anomalies from ECGs with 12 lead, enduring a few seconds to several seconds.

**Table 1:** According to the 'First label' annotations, the data outline for the training set is as follows from CPSC DATASET.

TYPE	#Footages	Stage Period (s)				
		Max	Min	Mean	Median	SD
PVC	672	60.00	6.00	20.21	15.00	12.85
PAC	556	60.0	9.00	19.46	14.00	12.36
LBBB	207	60.00	9.00	14.92	12.00	8.09
AF	1098	60.00	9.0	15.01	11.00	8.39
I-AVB	704	60.00	10.00	14.32	11.27	7.21
STD	825	60.00	8.0	15.13	12.78	6.82
STE	202	60.00	10.00	17.15	11.89	10.72
Ordinary	918	60.00	10.00	15.43	13.00	7.61
RBBB	1695	60.00	10.00	14.42	11.19	7.60
Overall	6877	60.00	6.00	15.79	12.00	9.04

The ECGs with 12-lead utilized in CPSC-2018 incorporate a typical sort of eight distinctive sorts that are definite as: AF- Atrial Fibrillation

(1) I-AVB- First-degree atrioventricular block
(2) LBBB- Left Bundle Branch
(3) RBBB- Right Bundle Branch Block
(4) PAC- Premature Atrial Contraction
(5) PVC- Premature Ventricular Contraction
(6) STD- ST-segment depression
(7) STE- ST-segment elevation

**Fig. 3:**
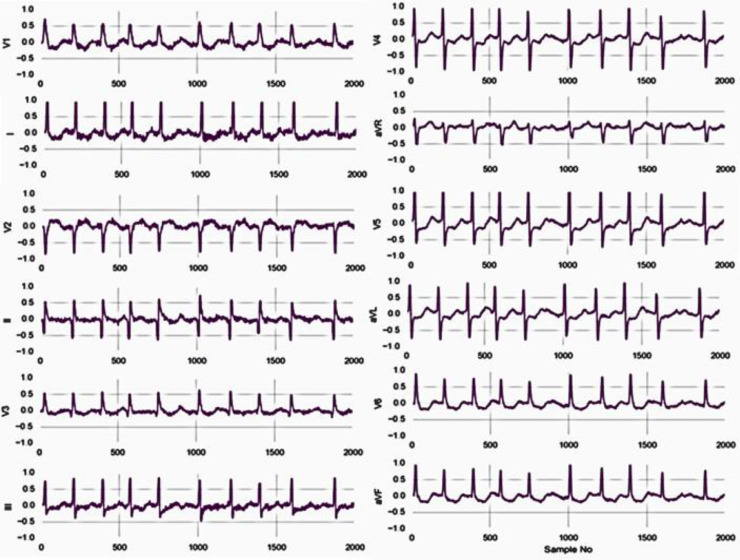
Waveforms of the ECG for 12 lead (http://2018.icbeb.org/Challenge.html).

Fig. 3 shows the waveforms of the 12-lead ECG. The anticipated system contains the dataset on which we have performed 3D wavelet change to eliminate the noise from the information or denoise the signals. In the wake of denoising the signals, we have to track down the pinnacle of the signs and take care of the information to SVM for double characterization. We have specified the signals through SVM, whether they are usual or unusual, assuming the label contains a strange class.

### QRS detection

**Fig. 4:**
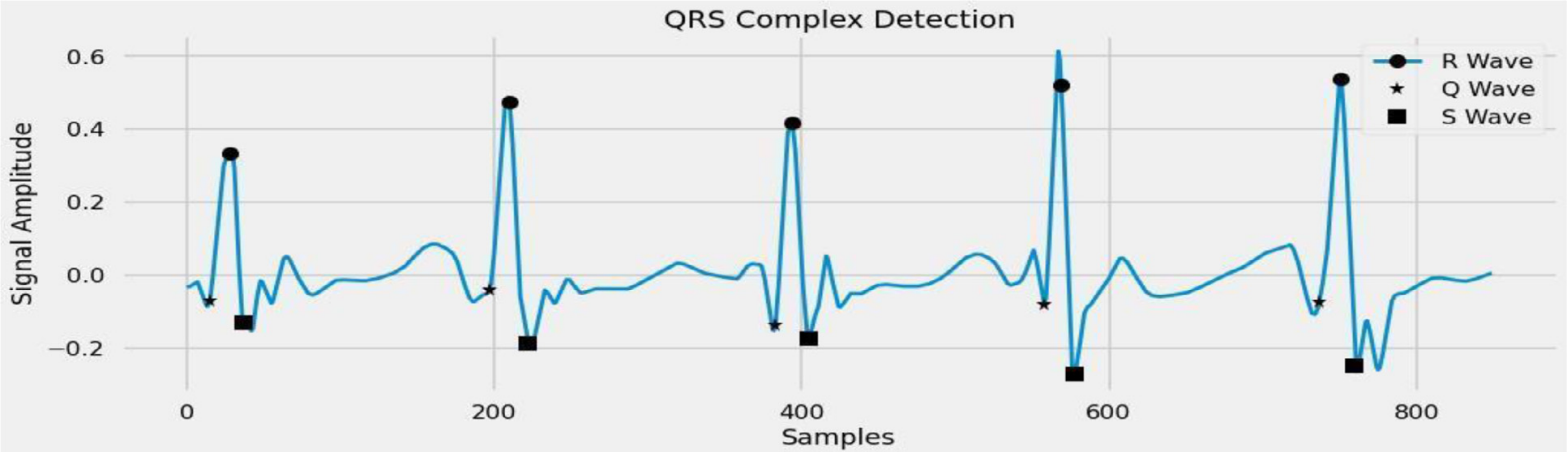
QRS detection in the ECG signals.

The ECG data blocks the beat-to-beat stretch by the QRS identification process. Therefore, the QRS is the central underlying part of the ECG.  When the heart muscle contracts during a heartbeat, this leads to what we termed as Ventricular Depolarization. The R wave is the pinnacle of the QRS complex, the time position of that pinnacle characterizes the hour of the heartbeat. The period between one R peak and the subsequent is called an RR span. For QRS distinguishing proof, we utilized the notable ECG k, a MATLAB tool compartment for ECG examination. The wave recognition procedure was utilized inside the ECG-unit structure. Fig. 4 shows the QRS value detection in the ECG signals. The RR interval sequences that results were recorded to maintain the square design.

### Wavelet (3D) transformation

A significant deprivation of the Fourier Transform is that it detects worldwide recurrence data, which means frequencies that persevere over a whole sign. This sort of sign decay may not work well for all applications, for instance, Electrocardiography (ECG) [12,13] where signals have short periods varying. An elective methodology is the Wavelet Transform, which deteriorates a capacity into a bunch of wavelets. The Wavelet is a wave-like swaying that is limited on schedule. A model is given beneath. Wavelets follow two fundamental properties: scale, and area. The s scaling (or enlargement) characterizes how "extended" or "crunched" a wavelet is processed. The property is identified with recurrence as characterized by waves. Area describes where the wavelet is located according to schedule.In the articulation above the Parameter "a" sets in the size of the wavelet. If we decline its worth, the wavelet will look more squished. This, in turn, can capture high-recurrence information. Conversely, the worth of "a" will extend the wavelet to catch low-recurrence data. The fundamental idea is to decide the amount of a wavelet if there is a sign at a given scale area. This is by large what convolutions are for the individuals who know about them. A sign is convolved at different scales with a bunch of wavelets. In another way, we select a wavelet of a particular scale. Then, at that point, we slide this wavelet through the entire sign, changing its area, and duplicating the wavelet sign at each time step. This multiplication's product gives us a coefficient for that wavelet scale at that time step. Finally, the wavelet scale is prolonged (for instance, the red and green wavelets), and the interaction is repeated.

**Fig. 5:**
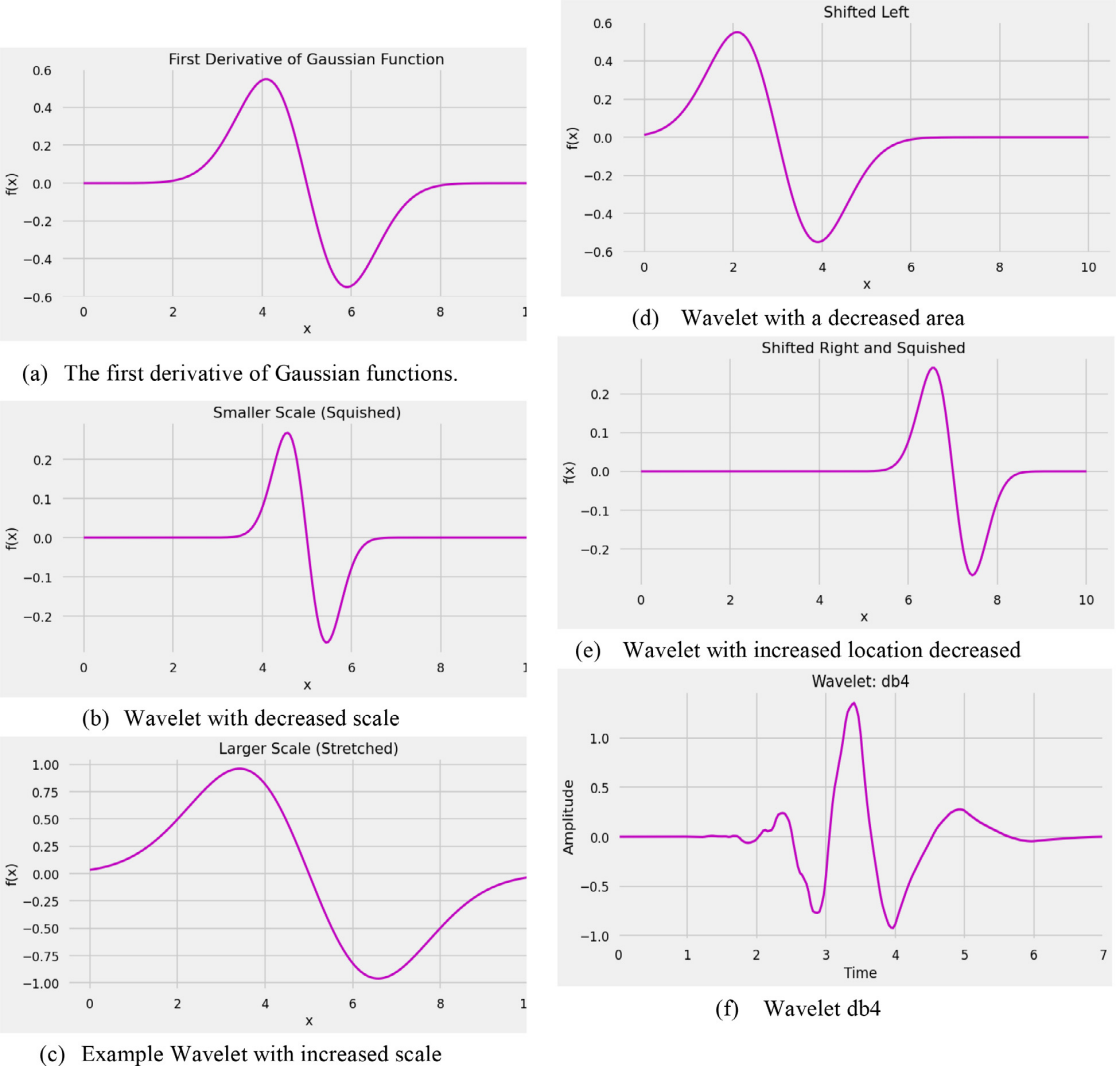

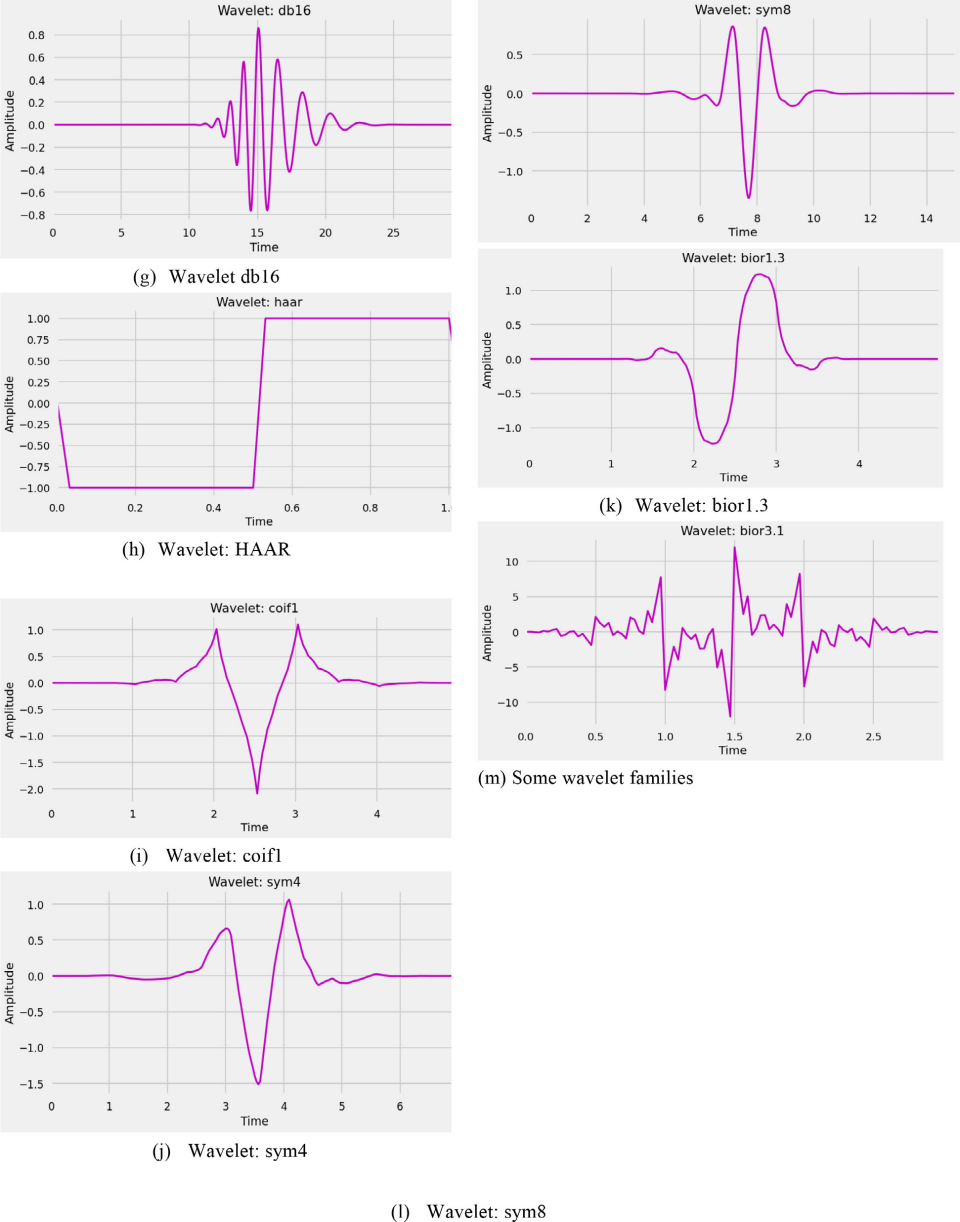
Wavelet Examples.

Wavelet Transforms are separated into two classifications: consistent discrete. The chart above shows the definitions for each kind. For example, the Continuous Wavelet Transform (CWT) utilizes each conceivable wavelet over an endless number of scales areas, the primary difference between these two variants. On the other hand, the Discrete Wavelet Transform (DWT) [14,15] utilizes a limited arrangement of wavelets characterized at specific scales. Different types of wavelet transformation are given in Fig. 5(a)–(m).

### Detecting peaks in ECG signals

**Fig. 6:**
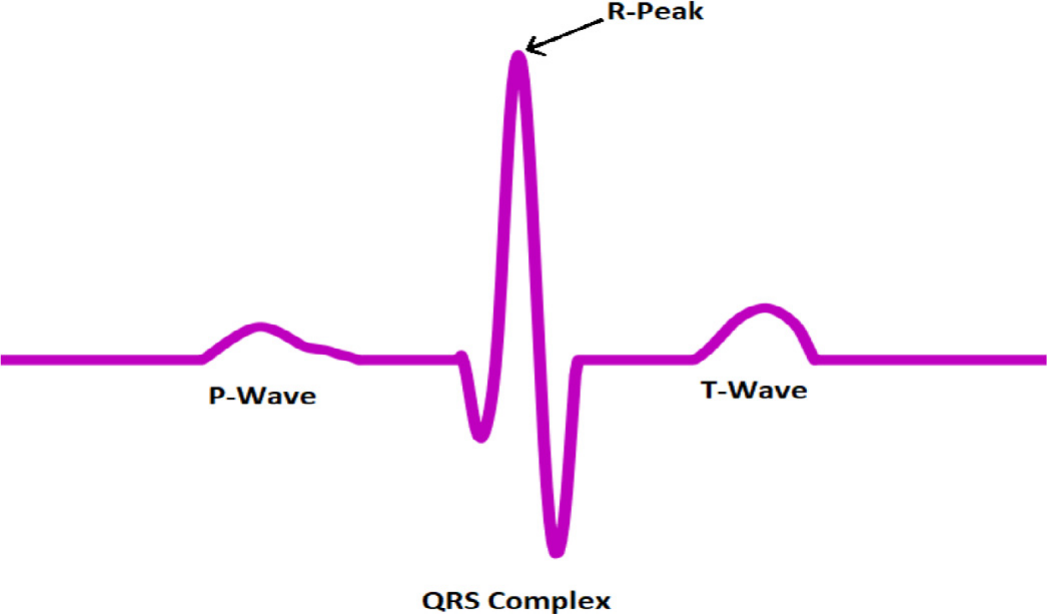
Sketch of a typical ECG signal resulting from the heartbeat.

For finding R-peaks [16] we utilize a sort of 3D discrete wavelet transformation to assist an electrocardiogram (ECG) which identifies heart movement, in this model. In an ECG signal [17], R-peaks usually are the most elevated. They are a part of the QRS complex, which is particularly wavering related to ventricular withdrawal atria extension. Computing pulse fluctuations are simpler when R-peaks [18] are distinguished heart rate variability (HRV). Fig. 6 depicts the sketch of a typical ECG signal resulting from the heartbeat

In reality, we hardly see ECG readings as clear as the one seen above. ECG data [19] is by large noisy, as displayed in this example. When used with crude information for R-peak identification, basic pinnacle revelation calculations will neglect to sum up. The wavelet change can help convert the sign into an arrangement that our pinnacle locator instrument can comprehend. We apply to extract R-peaks from the ECG waveform, using the maximal overlap discrete wavelet transform (MoDWT). The Symlet wavelet with eight evaporating moments (sym8) is utilized at seven particular scales.

### Hyper parameter tuning

A numerical model with different bounds obtained from the data is known as a machine learning model. However, there are some restrictions, or so-called hyperparameters, that cannot be purely scholarly. People ordinarily pick them up depending on some instinct or hit preliminary before the actual preparation starts. These boundaries display their significance by working on the presentation of the model, for example, its intricacy or its learning rate. Models can have numerous hyper-boundaries, observing all that blend of boundaries can be treated as an inquiry issue. SVM additionally has some hyper-boundaries (like what C or gamma esteems to utilize), and observing the ideal hyper-boundary is a tough assignment to settle. However, it tends to be found simply by attempting all mixes and seeing what boundaries work best. The fundamental thought behind it is to make a matrix of hyper-boundaries simply attempt their mixes in general (consequently, this technique is called Grid search). Grid Search CV takes a word reference that portrays the boundaries that could be occupied a stab with the model to prepare it. The network of limitations is categorized as a word reference, where the keys are the limitations and the abilities are the backgrounds to be tried. To work on the model precision, a few boundaries should be tuned. Three significant boundaries include

Kernels: The fundamental capacity of the part is to take low-layered information space and change it into a higher-layered space. It is, for the most part, valuable in non-direct partition issues.

C (Regularization): C is the punishment boundary, addressing misclassification or blunder terms. The blunder term shows the SVM – ‘Amount of mistake that is endurable. This is how you can compromise between choice limit misclassification terms. When C is high, it will characterize every one of the information accurately; likewise, there is an opportunity to overfit.

Gamma: When gamma is larger, nearby focuses have a greater influence; when gamma is lower, far away focuses are also taken into account when computing the decision limit of the conceivable line of detachment.

### Architecture of SVM

Hyperplane - A hyperplane is a decision restriction distinguishing the two SVM [20,21] classes. Different classifications may be assigned to data points on each side of the hyperplane. The hyperplane depends upon the number of information features in the dataset. Expecting we have 2 data points the hyper-plane will be a line. Besides, expecting that the number of components is 3, it will be a two-layered plane.

Support vectors- The information or data points that are contiguous to the hyper-plane are support vectors [22]. Therefore, we need to choose a hyperplane, for which the edge, i.e., the distance between the help vectors hyper-plane, is most extreme. Indeed, even a negligible impedance in these help vectors can change the hyperplane.

Maximal edge classifier (MEC) - A hypothetical classifier that best explains SVM capacities’ working is termed a MEC. The numeric data factors (x) in your data (the segments) structure an n-layered space. E.g., expecting you had 2 data variables would shape a 2-layered space.

The SVM is a management (preparation) system used to organize different data (information) gatherings. The estimator seeks the optimal separation surface, which is why this technique is used. The classifier's goal is to discover a hyperplane that can divide data bundles with the ultimate objective that the distance between the mean of the things in the hyperplane is generally outrageous. In this manner, SVMs are moreover called Maximal Marginal Classifiers. The information is processed by separating into testing and training datasets, then, at that point, utilized for arrangement. The information utilized for grouping comprises chosen highlights; the data is parted into getting-ready testing sets that are then cast off for request. The information utilized for order comprises chosen highlights, and a comparing objective worth, also called the class mark. The chosen highlight vector is given to contribute to the SVM model, and a comparing objective worth is anticipated. Numerical capacities, for example, Kernels, are utilized by the classifier. The portion can accept information as info changes straightly indivisible information to directly distinct ones. In this review, the portion of work utilized is the Gaussian Radial Basis work, and the classifier is executed utilizing Library for Support Vector Machines (LIBSVM).

**Fig. 7:**
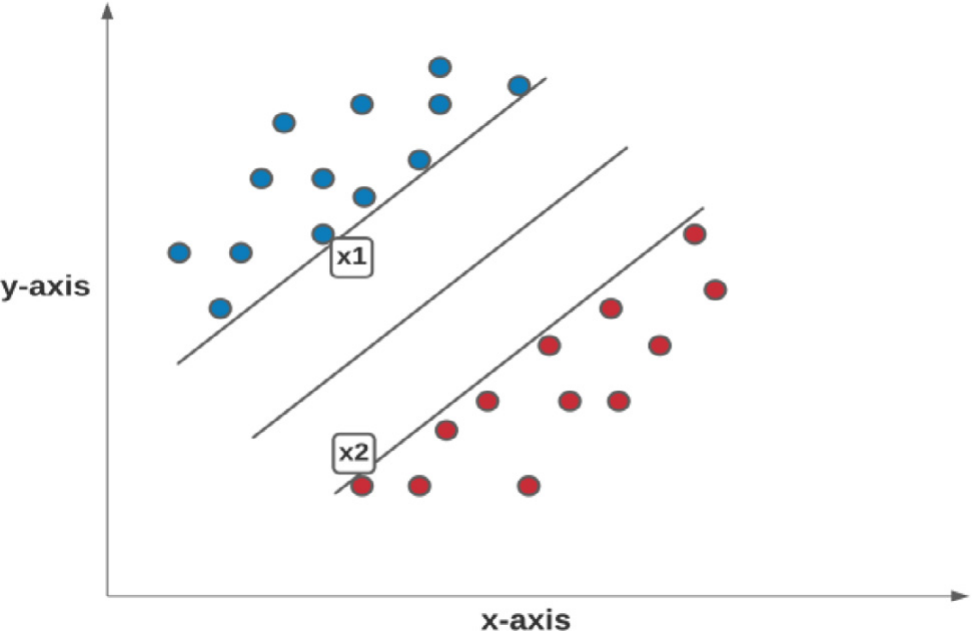
Architecture of the SVM model.

This portion counts on Gamma limit (γ) Regularization consistent (C). To get the best show of the SVM classifier, the ideal group of the regularization consistent (γ) and (C) should exist. Consequently, a matrix hunt with profoundly creating progressions was executed. Each mix takes a stab at utilizing cross-approval. The limits with the best cross-endorsement precision are now selected. In Fig. 7 blue red color specifies the two classes of data x1, and x2 are the data points of the classes respectively

## Results and analysis

Electrocardiograms (ECG) [23,24] are broadly utilized for the analysis of cardiovascular arrhythmias. We examine the utilization of machine learning arrangement calculations for ECG examination arrhythmia recognition. Two classes, Normal (N) and unusual (Ab) are at the same time introduced to SVM classifier [25]. The ECG signals are discovered in the CPSC 201,812-lead Arrhythmia Database. The Waveform the waveform database (WFDB) Software bundle is utilized to read the database explanation data and track down the R (peak) zone of the ECG Wave (P wave, QRS wave, T wave). The 3D wavelet change is utilized to extract highlights. This ensures a modest decrease in dimensionality and the information vector for the classifier is de-noised. ECG beats order is achieved utilizing SVM [26] with preparing test datasets. Hyperparameter tuning Grid Search CV is utilized for seeing the best boundary upsides of the SVM. The point of the review is to set up the benefits of SVM for distinguishing various sorts of arrhythmias based on multi-lead accounts following sign pressure in the Fourier space. Execution of the calculations was performed in PYTHON. The arrangement exactness of the anticipated plot is 98.25% utilizing SVM. The result examination begins with setting up the disarray network dependent on the approval results. Underneath the figure shows the disarray network as far as the number of beats with a valid and anticipated mark: N anticipated name, real name. The anticipated mark was set up with an SVM characterization model. A ROC bend shows what the edge level means for the paired classifier's symptomatic capacity. The region beneath the ROC bend demonstrates the classifier's comprehensive exhibition, i.e., a region more like 1 demonstrates a superior characterization performance. The underneath figure shows the disarray grid for SVM before the hyper boundary, which has a few issues as we have not done the hyper boundary tuning.

### Confusion matrix

The confusion matrix is the representation of the (N x N) matrix which is applied to assess the performance of the existing classification model, in which ‘N’ denotes the number of target classes. The matrix examines the actual predictions made by the machine learning model.

**Fig. 8:**
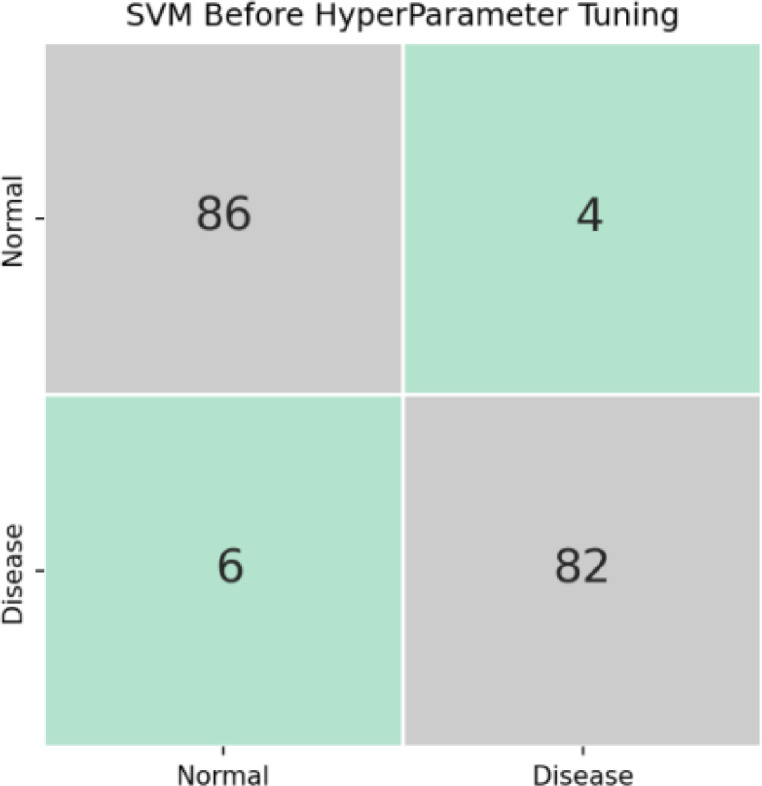
SVM's Confusion matrix before hyperparameter tuning.

**Fig. 9:**
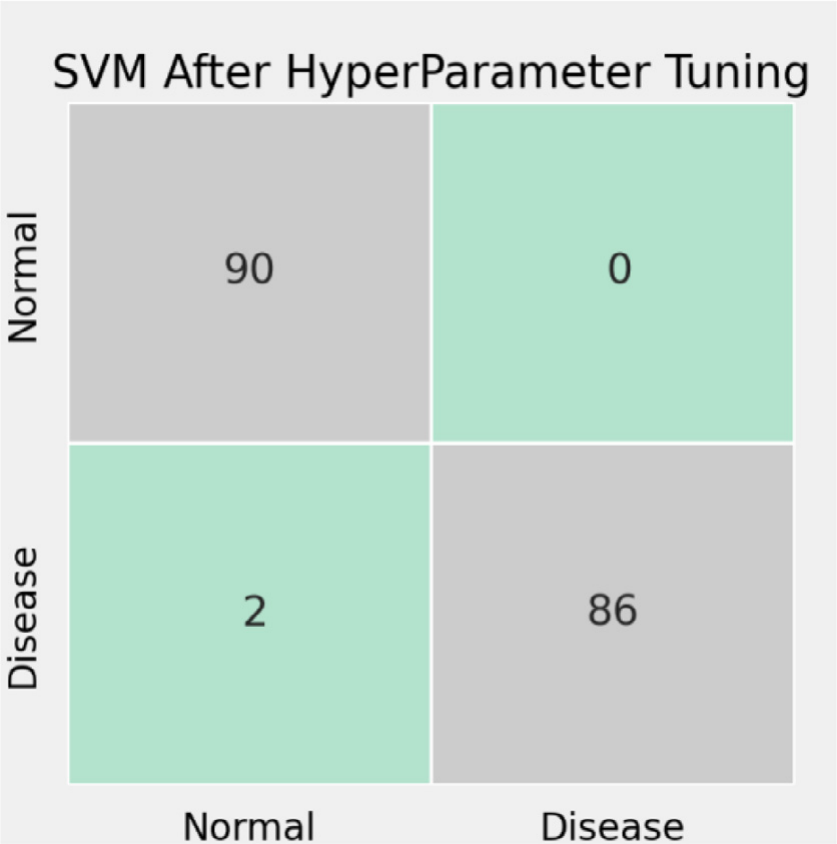
SVM's Confusion matrix after hyperparameter tuning.

Fig. 8. shows the SVM's confusion matrix after the hyperparameter, which has resolved the issues presently before. The reason for this study analysis was to improve the binary classification accuracy that is feasible for further development when utilizing a multi-lead database as a contribution to the classifier. In Fig. 9 the anticipated calculation accomplished order exactness of 98.88%. Also, these outcomes Other ECG beat classifications have been compared frameworks, for example, CSVM is 98%, and SVM with wavelet variation is almost all the way. The utilization of SVM multi-lead information bases gives better order and conditions of recognition rate than other revealed studies.

### ROC curve

**Fig. 10:**
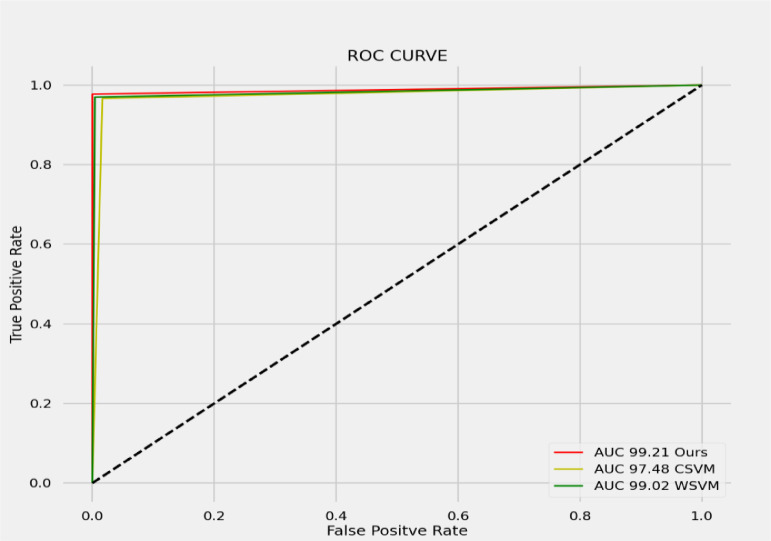
Comparison graph of our method with CSVM and WSVM.

The receiver operating characteristic (ROC) curve shows the actual favorable false-positive rates at different order limits, though the under the curve (AUC) shows an overall calculation of a machine learning model's presentation transversely all classification stages. In the predictive model, understanding between the true positive rates and the false positive rate utilizing distinctive probabilities thresholds are summarized by ROC Curves. In a predictive model, precision-recall includes the compromise between the positive rate and the positive predictive value with variable likelihood edges. Fig. 10 depicts the comparison graph of our method with CSVM and WSVM.

The present assessment confirmed that applying ECG rhythm signals from 12 leads might trigger greater categorization results with high accuracy further developing the speculation capacity of the SVM classifier. Multi-layered SVM with Clifford Algebras will be regarded for multi-lead signal analysis in no time, since it might give a more extensive, precise, and adaptable ECG beat classification plot, to execute the multi-lead signal analysis. The anticipated method will also be expanded by using a flexible wavelet, where a different wavelet work is inferred at each decreasing level to produce an increase in the input vector, with the aim of having the information vectors for the classifier denoise wavelet transformation coefficients. Higher-order moment strategies are used in the adaptive wavelet filtering method. We have applied a downsampling approach in our methodology to get better predictions from other approaches and hence it is proved with the help of downsampling techniques and 3-d wavelet transformation we get the accuracy of 99.21% of our method. The reason for this study analysis was to set up and improve the binary classification accuracy that is feasible for further development when utilizing a multi-lead database as a contribution to the classifier. Table 2 lists the proposed calculation accomplished an order exact-ness of 98.88%. Also, these outcomes have been contrasted and other ECG beat classification frameworks, for example, CSVM [27] is 98%, SVM with wavelet variation WSVM [28] is almost all the way. The utilization of SVM and multi-lead information bases gives better order and brings conditions of recognition rate than other revealed studies. The below table shows the comparative analysis based on the different approaches used by other methods and our method.

**Table 2:** Accuracy of the proposed approach.

Method	Approach	Accuracy
Our Method	3-D wavelet transformation	99.02%
CSVM	DFT	99%
WSVM	DWT	97.48

### Classification report

Aclassification report is a performance assessment metric in machine learning. It is used to demonstrate the precision, accuracy, F1 Score, and support of the classification model you constructed. There are four methods for figuring out whether the forecasts are right or wrong: Accuracy is the classifier's capacity to avoid naming (labeling) a negative event as positive.It is characterized as the proportion of true positives to the amount of valid false negatives of each class:

(1) TP – True Positives, FP – False Positives.…………………………

Precision – Accuracy of optimistic predictions.

(2) Precision = TP/ (TP + FP) …………..……….……… .

Recall – Define the percentage of cases/instances that you caught.

A classifier's capacity to locate all positive occurrences is defined as ‘Recall’. The ratio of genuine positives to the sum of true positives and false negatives is termed a class.

FN – False Negatives, Recall the Fraction of positives that were properly recognized.

(3) Recall = TP/(TP+FN) …………………………………

F1 score – Depicts the optimistic predictions that are correct.

(4) F1 Score = 2*(Recall * Precision) / (Recall + Precision) ……………….

TRUE Negative (TN): The case was predicted to be negative.

TRUE Positive (TP): The case was effective and was expected to be preferred.

FN (False Negative): The case was effective, but the outcome was negative.

False Positive (FP): The case was negative, but the prediction was positive

**Fig. 11:**
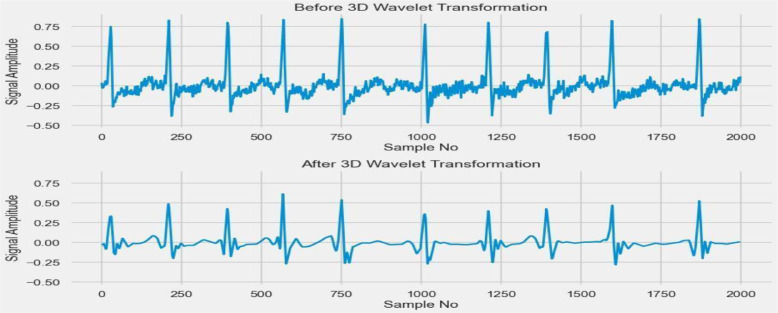
Preprocessing before 3D wavelet after wavelet transformation.

Table 3 lists the SVM classification report before the hyperparameter. Table 4 lists the SVM classification report after hyperparameter tuning. Fig. 11 shows the ECG signal [29] after the 3D wavelet transformation i.e., after denoising the signal.

**Table 3:** SVM classification report before hyperparameter tuning.

	precision	recall	f1-score	assist
0	0.934783	0.955556	0.945055	90
1	0.953488	0.931818	0.942529	88
accuracy	0.94382	0.94382	0.94382	0.94382
macro avg	0.944135	0.943687	0.943792	178
weighted avg	0.94403	0.94382	0.943806	178

**Table 4:** SVM Classification Report after hyperparameter tuning.

	precision	recall	f1-score	Support
0	0.978261	1	0.989011	90
1	1	0.977273	0.988506	88
Accuracy	0.988764	0.988764	0.988764	0.988764
macro avg	0.98913	0.988636	0.988758	178
weighted avg	0.989008	0.988764	0.988761	178

The graph shows the result of pre-processing. We opted for the 3D wavelet transformation of data to detect and dilute the noise present in the dataset.

### Detecting R-peak graph

**Fig. 12:**
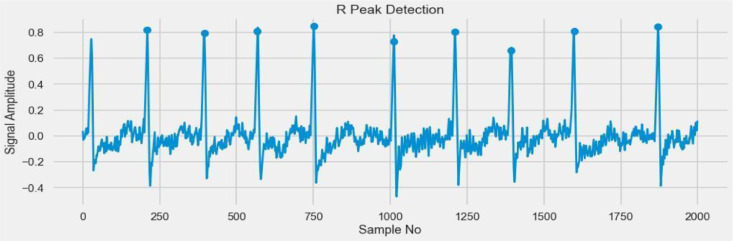
R-Peak graph without denoise to present noise.

**Fig. 13:**
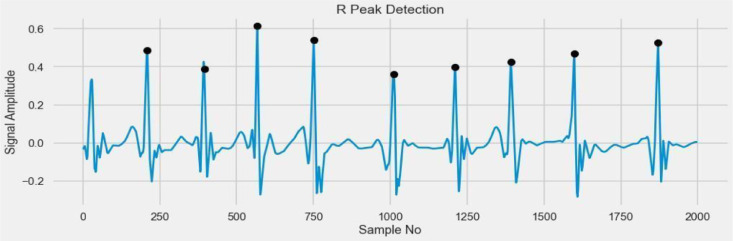
R-Peak graph without denoise and present noise diluted.

The R-peak time in a particular ECG [30] lead is the span from the earliest beginning of the QRS, preferably determined from different while recorded leads, to the peak(limit) of the R wave or R' if present. Fig. 12 shows the R peak graph without denoise to present noise in the dataset, and Fig. 13 shows the R-peak graph without denoise and present noise diluted.

#### R-R interval

**Fig. 14:**
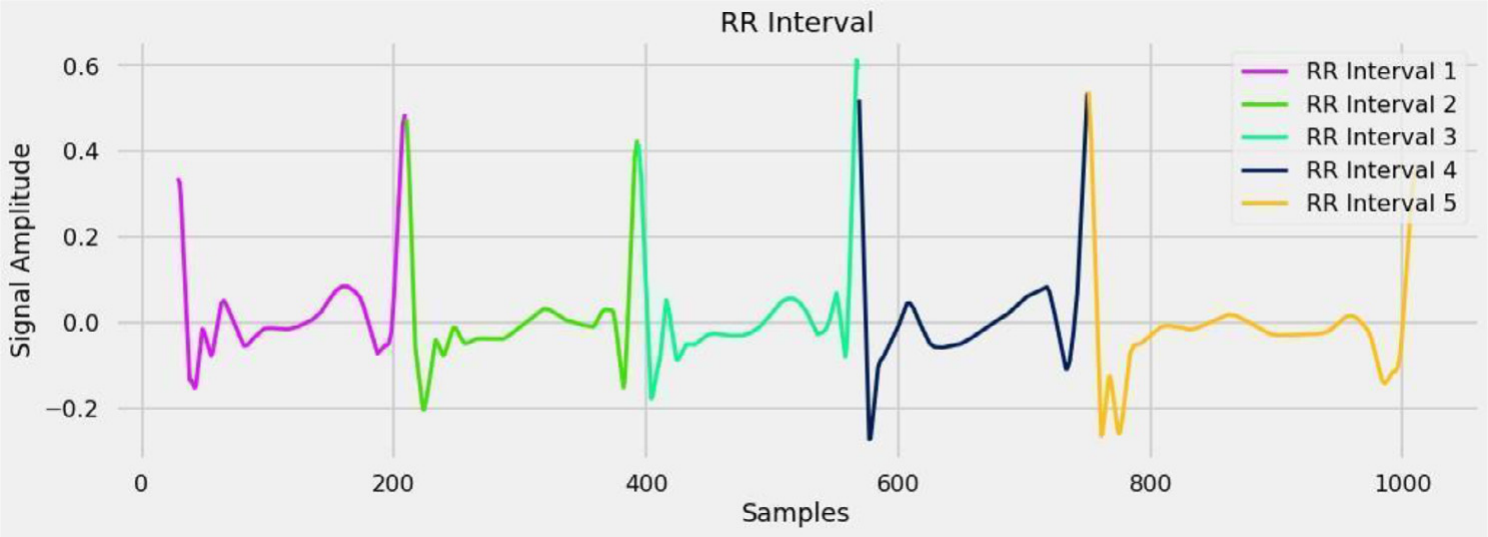
R-R interval in the ECG signal.

RR interval, the time passed between two progressive R-rushes of the QRS signal on the electrocardiogram (it is equal, to the HR) is an element of natural properties of the sinus nodes just as autonomic impacts. An ECG Signal's R-R Interval: Fig. 14 shows the R-R interval in the ECG signal.

## Limitations

Multiple numbers of patients' ECG signals were recorded. However, the signals range from 6 to 60 s. To obtain this, more comprehensive data in sequence are required. A mass number of patients' ECG signals were recorded. In slightly case, the signal endures for 6 to 60 s. Longer data intervals are prerequisite to test and maybe update arrhythmia identifying value. One more inconvenience of the review is that the RR intervals were recovered from ECG, connected to the accessible information source. Due to the financial expense foundation necessities of ECG [31,32] recording, deciding the RR spans is probably going to change in a functional setting. Various methodologies are utilized to set up the RR intervals in financially savvy pulse screens, for example, sensors worn on the chest wrist. Thus, we want to approve the information assortment process before applying our discoveries to plan viable frameworks that work in clinical practice. The speculated surplus RR intervals fill in as a calming update that physiological sign healing may be inclined to botches. Since QRS detection [33,34] is not an exemption from this rule, RR interval signals might contain botches. It is unfeasible would adjust the sign understanding outcomes to address these errors by visual evaluation by a human expert [35]. The vast volumes of information that should be confirmed would make it unrealistic [36]. This verification process would considerably impact any issue arrangement depending on RR interval signals. Therefore, a practical framework for recognizing arrhythmias should have the option of becoming familiar with RR interval signal anomalies; this limit should be specified throughout the design phase.

## Conclusions

SVM with hyperparameter tuning based on DWT was used to create an ECG data categorization framework for organizing the various beat patterns. The use of reliable long-length records to extract distinctive ECG characteristics highlights the SVM classifier model has been enabled by the CPSC 2018 ECG signals. Symlet-8 signals, on the other hand, produced superior results with great correctness. The classification results suggest that by increasing the information vector of the preparation information, the anticipated model can also accomplish high affectability when grouping ECG beats into distinct arrhythmia types. The model was verified utilizing the ECG signals captured throughout the test. The SVM classifier's average precision for improved noise reduction and lower frequency noise filtering using DWT. The average precision accomplished for recorded signals is 99.2 percent. Patients' medical problems can be split down and rely on the results achieved from the classifier. The trial may go on longer if ECG signals from patients with arrhythmia who were using the device were included, or if the product was coordinated with the equipment. Future research could simplify the computing environment and increase setup efficiency.

In this paper, we presented the SVM model for binary classification for arrhythmia order. Later on, we want to decide how we can arrange abnormal classes in the other eight categories (disease) models in a viable clinical choice help situation. Such a review could give further knowledge into the job of standard information for arrhythmia location. Still, in addressing the limitations outlined in the previous section, more comprehensive measurement data is needed. Furthermore, fuzzy logic for QRS identification may assist with decreasing errors and subsequently work on the viable importance of the anticipated arrhythmia discovery strategy.

### Ethics statements

The methods used in the study did not involve any human or animal subjects. No data was used or collected for this work.

### Data availability

No specific data is used. The open-source data is used in the work

The China Physiological Signal Challenge 2018: Automatic identification of the rhythm/morphologyabnormalities in 12-lead ECGs http://2018.icbeb.org/Challenge.html https://www.kaggle.com/datasets/bjoernjostein/china-12lead-ecg-challenge-database

## CRediT authorship contribution statement

**Shikha Dhyani:** Conceptualization, Methodology, Software. **Adesh Kumar:** Writing – review & editing, Supervision. **Sushabhan Choudhury:** Visualization, Writing – review & editing, Supervision.

## Declaration of Competing Interest

The authors declare that they have no known competing financial interests or personal relationships that could have appeared to influence the work reported in this paper.
